# 3dRNA: Building RNA 3D structure with improved template library

**DOI:** 10.1016/j.csbj.2020.08.017

**Published:** 2020-08-28

**Authors:** Yi Zhang, Jun Wang, Yi Xiao

**Affiliations:** Institute of Biophysics, School of Physics, Huazhong University of Science and Technology, Wuhan 430074, Hubei, China

**Keywords:** RNA 3D structure prediction, 3D template library, 3dRNA

## Abstract

Most of computational methods of building RNA tertiary structure are template-based. The template-based methods usually can give more accurate 3D structures due to the use of native 3D templates, but they cannot work if the 3D templates are not available. So, a more complete library of the native 3D templates is very important for this type of methods. 3dRNA is a template-based method for building RNA tertiary structure previously proposed by us. In this paper we report improved 3D template libraries of 3dRNA by using two different schemes that give two libraries 3dRNA_Lib1 and 3dRNA_Lib2. These libraries expand the original one by nearly ten times. Benchmark shows that they can significantly increase the accuracy of 3dRNA, especially in building complex and large RNA 3D structures.

## 1 Introduction

Three-dimensional (3D) structures of RNAs play important role in performing their functions [1]. For example, for ribozymes, we must find out how their active centers combine and react with the substrates to truly understand the catalytic mechanism. To do this, the experimental methods like X-ray crystallography, nuclear magnetic resonance (NMR) spectroscopy, and cryo-electron microscopy to determine RNA structures are still challenging and laborious currently. Faced with a large number of RNA sequences, another way to build or predict RNA 3D structures is through computational approach [2].

Earlier RNA 3D structure prediction methods, such as ASSEMBLE [3], YUP [4] and MANIP [5], all need human intervention and adjustment. For example, although ASSEMBLE provides users with an interactive graphical interface to analyze and predict RNA, all interactions including base pairing and base stacking need to be manually annotated. After continuous developments, more and more automated prediction programs have been proposed [6], [7], [8], [9], [10], [11], [12], [13], [14], [15], [16], [17], [18], [19], [20], [21], [22], [23], [24], [25], [26], [27], [28], [29], [30], [31], which can be roughly divided into two categories. The first category is ab initial prediction based on molecular dynamics simulation. For example, iFoldRNA [16] adopted an 3-beads RNA model and highly-efficient discrete molecular dynamics simulation method in order to quickly search the possible conformation space; NAST [6] used a coarse-grained statistical potential and a simple molecular dynamics algorithm to conduct conformational sampling under secondary structure and other constraints. The recently proposed three-bead CG model [29], [30], [31] with involving an implicit electrostatic potential and sequence-based thermodynamic parameters can simultaneously predict 3D structures and stability of RNAs in ion solutions. The model give reliable predictions on 3D structures and stability for RNA hairpins [5], double-stranded RNAs, and RNA pseudoknots after strict verification. However, due to the need for huge computational power to sample the conformational space, the use of ab initio methods is rather limited to smaller molecules. The second category is template-based approach, most of which have no restrictions on RNA size, but rely on the database of experimentally solved structures. For example, FARNA [14] / FRAFRA [15] uses Monte Carlo method to randomly select 3D structures of 3nt fragments from a template library extracted from the ribosomes to assemble RNA 3D structures; RNAComposer [17] selects the 3D structures of fragments from FRABASE database built in advance [18], [19], assembles them together to form a complete structure, and then optimizes this assembled structure in dihedral angle space and Cartesian space; Recently proposed VfoldLA [20], [21] is different from the previous template-based methods in the way of template searching and it only searches for the templates for single strands of loops/junctions instead of the entire loop motif from the template library and its template matching rate and prediction efficiency is higher; 3dRNA [22], [23], [28] proposed in our laboratory can automatically predict 3D structure of an RNA by assembling 3D templates of Smallest Secondary Elements (SSEs) [20], including stem, hairpin loop, bulge loop, internal loop, open loop and junction. The prediction accuracy of 3dRNA is about 3 Å for RNA less than 50nt and 6 Å for RNA of 50-100nt. However, due to insufficient number of the templates in the original 3D template library, it is difficult for 3dRNA to predict RNA 3D structures with longer chain and/or complex topology.

In this paper, we report new 3D template libraries of 3dRNA improved by using two different schemes: 3dRNA_Lib1 and 3dRNA_Lib2. Compared with the old library (called 3dRNA_Oldlib), we mainly made the following improvements: (1) To enrich templates in the library, instead of retaining only non-homologous RNA monomer structure like in 3dRNA_Oldlib, the chains from different RNAs with the same sequence and secondary structure are reserved. (2) All modified nucleotides are retained by mutating them into standard ones. (3) All base pairs (including all non-standard base pairs) calculated by X3DNA [32] are reserved to obtain more accurate RNA secondary structures. (4) Single base pair (helix with one base pair) is preserved in the template library 3dRNA_Lib2. It is opened in 3dRNA_Lib1and 3dRNA_Oldlib. (5) 3dRNA_Lib1 and 3dRNA_Lib2 can be automatically updated.

## 2 Methods and materials

**Fig. 1:**
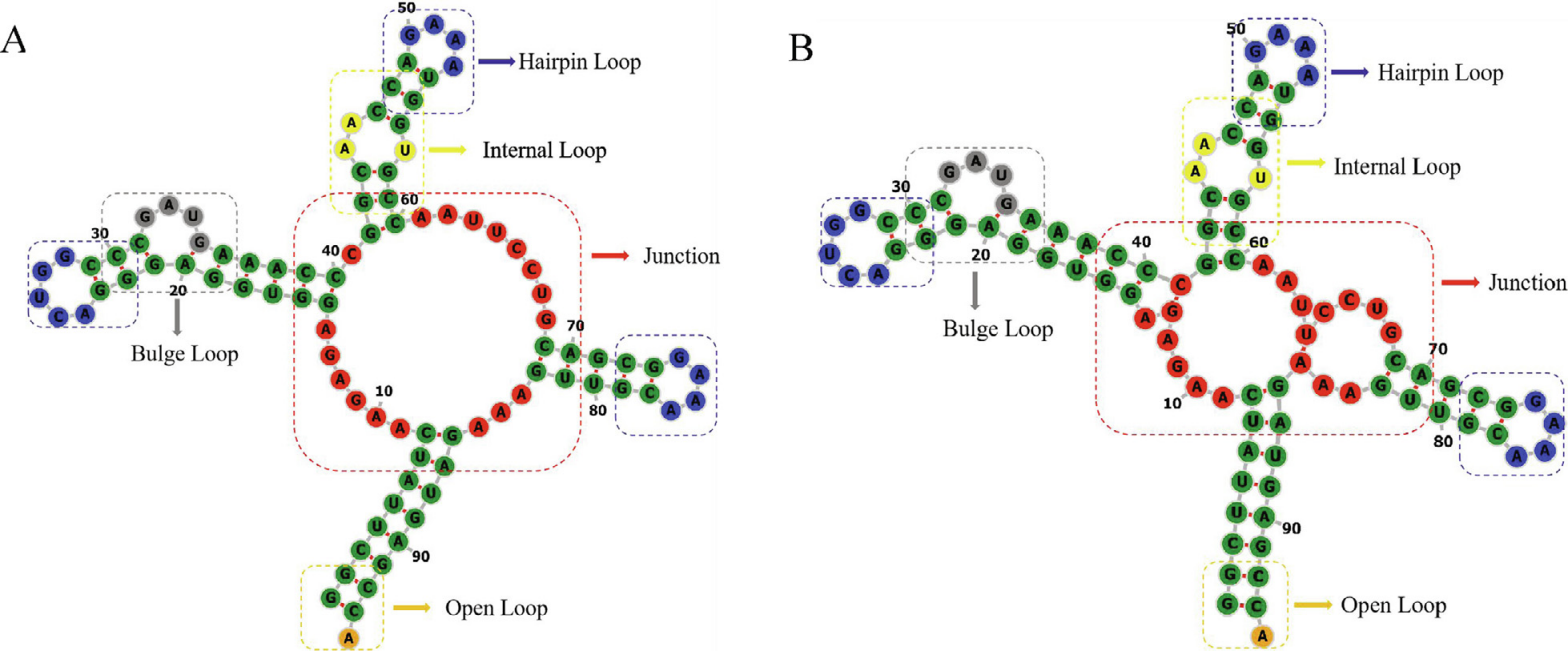
Definition of SSEs in 3dRNA for an RNA (PDB id: 2GIS). Stems are shown in green, hairpin loops in blue, internal loops in yellow, bulge loop in grey, open loop in orange, and junction in red. (A) and (B) shows the difference between definitions of an SSE in (A) 3dRNA_Lib1 and in (B) 3dRNA_Lib2 when there are cases of a single base pair (helix with one base pair) (here 13–41 and 64–85). In 3dRNA_Lib1 the single base pair is opened before identifying SSE. (For interpretation of the references to color in this figure legend, the reader is referred to the web version of this article.)

The 3D template library of 3dRNA is constructed by decomposing RNA molecules with known 3D structures into SSEs. The SSEs are defined as stem and different kinds of loops together with two base pairs of each stem connected with them, (see Fig. 1). The loops include hairpin loop, bulge loop, internal loop, open loop and junction, the most common base pairs are AU, GC and GU and they are called as standard base pairs in this work. Non-standard base pairs are also preserved. Two different template libraries (3dRNA_Lib1 and 3dRNA_Lib2) are constructed. Their difference is in the treatment of single base pair (helix with one base pair). In 3dRNA_Lib1 single base pair will be opened while in 3dRNA_Lib2 it will be reserved.

**Fig. 2:**
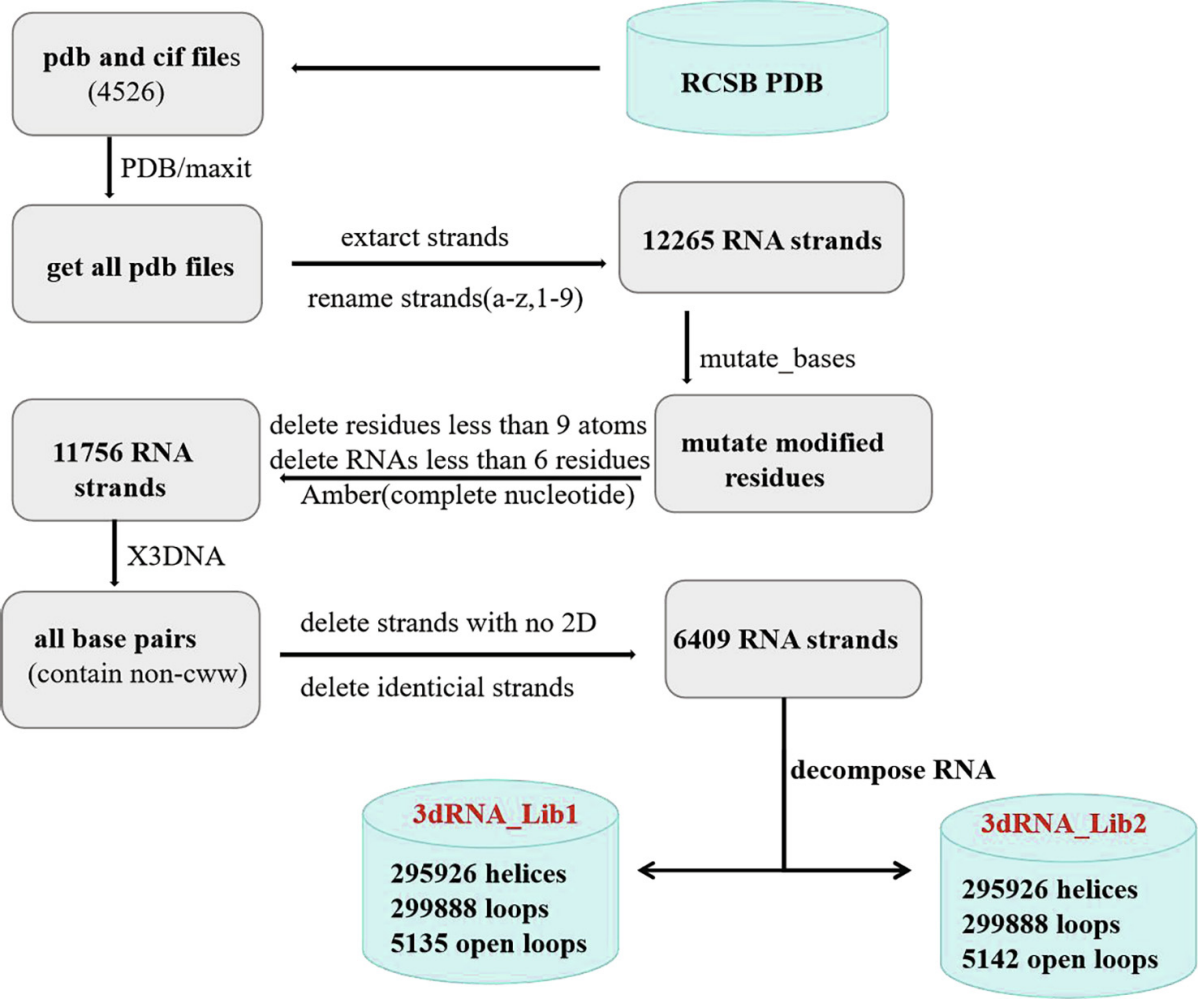
The procedure of building 3D template library of 3dRNA.

The construction of the 3D template library mainly includes PDB filtering and chain splitting, secondary structure calculation and SSE module decomposition. The detailed construction process of the 3D template library is shown in Fig. 2.

**PDB filtering and chain splitting.** We searched the RCSB PDB database [33] to download all the structures that contain RNAs and obtained 4526 RNA structures, including PDB format and CIF format. For the convenience of subsequent calculations, the MAXIT program in the PDB library was used to convert CIF file into corresponding PDB file. 3dRNA only predicts 3D structures of RNA monomers and so we extracted all of RNA single stands and obtained 12,265 RNA monomer structures. In order to ensure that all nucleotides are standard A, U, C and G bases, we used MUTATE program of X3DNA to mutate modified nucleotides to standard ones. Due to the missing of atoms of some nucleotides in certain measured structures, we eliminated the nucleotides less than 9 atoms and then used AMBER [34] to complete these nucleotides. RNA monomers less than 6 nucleotides cannot form SSE and are also deleted. Finally, 11,756 RNA monomer structures remained.

**Secondary structure calculation.** In order to decompose these 11,756 RNAs into SSEs, we also need to obtain their secondary structures. Here X3DNA is used to do this. Furthermore, we retain not only the standard base pairs (AU, CG and GU) but also all non-standard base pairs. Single-stranded RNAs without secondary structures are removed and for identical strands one of them is retained. Finally, 6,409 RNA monomers remained in this step.

**SSE decomposition.** According to the secondary structures of 6409 RNA monomers, their 3D structures are split into 3D templates according to the SSEs. These templates and their related information are put into the template library of 3dRNA. In order to facilitate the template searching in the library, the relevant information of the 3D templates of each SSE includes its sequence, dot-bracket notation, length and family. The decomposition adapts two schemes and so results in two different libraries: 3dRNA_Lib1 and 3dRNA_Lib2. In 3dRNA_Lib1 single base pair will be opened as in 3dRNA_Oldlib. This will decrease the accuracy of SSE secondary structure but increase the tolerance in template selection. In 3dRNA_Lib2 single base pair will be preserved to ensure the accuracy of SSE secondary structure. The final numbers of helices and loops in the template library 3dRNA_Lib1 and 3dRNA_Lib2 are the same, except the dot-bracket representations of the secondary structure of the SSEs in which single base pair is opened in 3dRNA_Lib1.

**Fig. 3:**
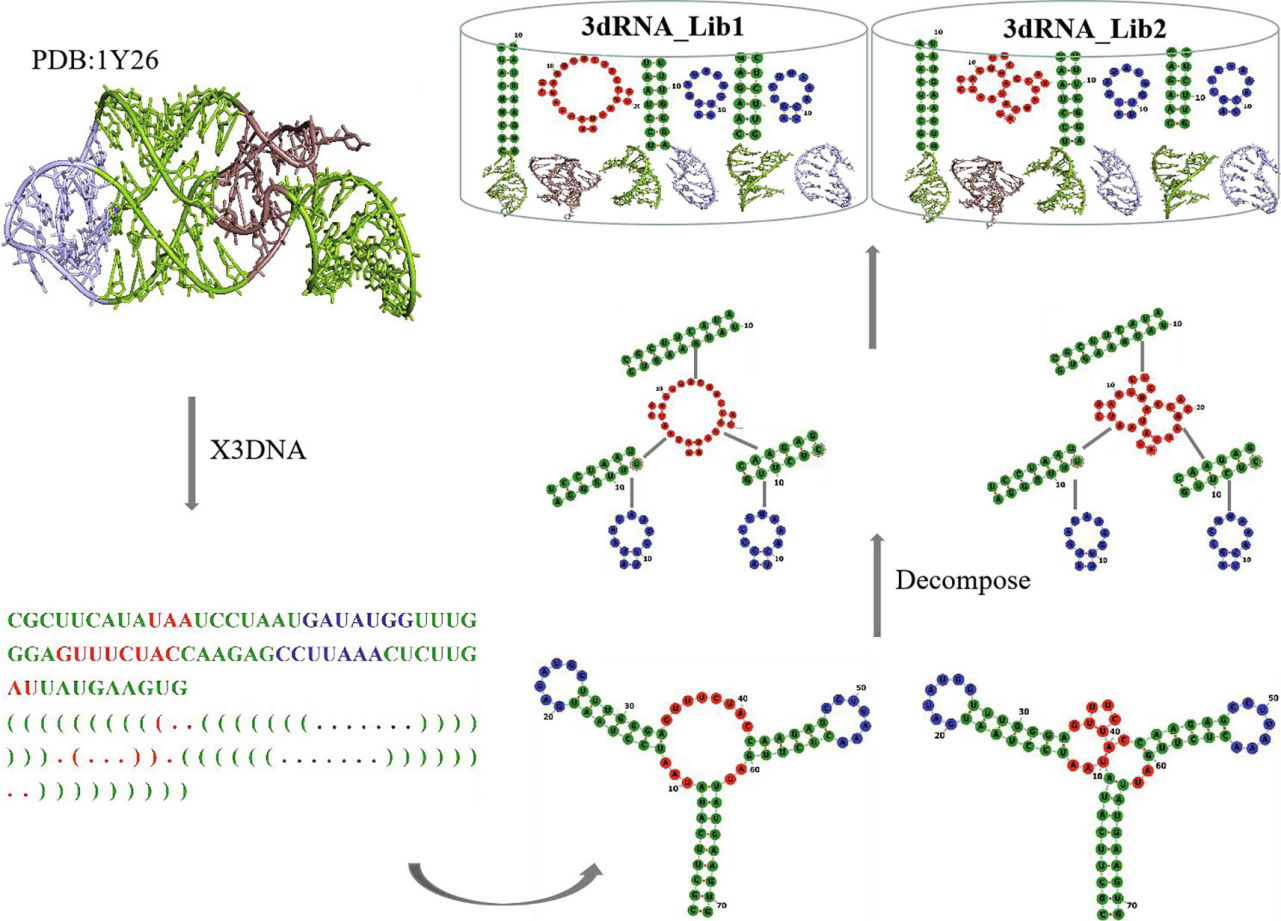
The flow of adding SSE 3D structures of an RNA (PDB id: 1Y26) to the template library. RNA 2D plots are generated using Forna [38].

As an example, Fig. 3 shows the process of adding SSE structures of 1Y26 to the template library. We firstly extract the base-pair information from the PDB-deposited RNA structure file, 1Y26.pdb. 3dRNA deals with RNA sequences with standard A, U, C and G and represents their secondary structures in ‘dot-bracket’ notation. All base pairs are first calculated by X3DNA. When a residue is paired with multiple residues at the same time, we consider the following criteria to filter out incorrect base pairs: whether the sequence interval of paired bases is greater than 4, whether this base pair is standard one, and whether a base pair is formed before and after this base pair. Having the secondary structure, we decompose it to different SSEs. For 3dRNA_Lib1 single base pair is opened and for 3dRNA_Lib2 it will be reserved. For example, in 3dRNA_Lib1 10–40 base pair and 35–39 base pair in 1Y26 are opened to get a large loop. Finally, according to the SSEs, the 3D and 2D structures of each SSE are added to the template library.

## 3 Results

**Fig. 4:**
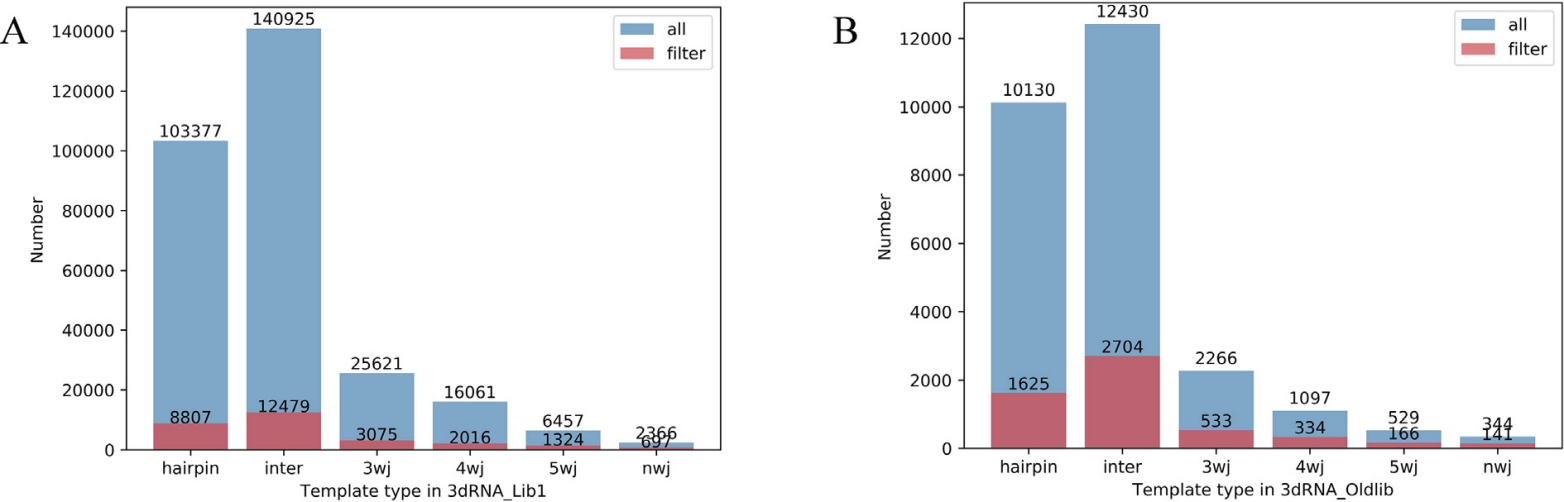
Statistics of different types of loops in 3dRNA_Oldlib (A) and 3dRNA_Lib1 (B)**. ‘**nwj’ represents junctions containing at least 6 helices, ‘all’ represents the number of loops of all types in the template library, and ‘filter’ represents the number of loops that keeps one of the SSEs with identical sequences and secondary structures (pseudoknots are ignored).

In comparison with about 50,000 templates in 3dRNA_Oldlib, the library 3dRNA_Lib1 has now been expanded by about 10 times. Fig. 4A and Fig. 4B show the statistics of different types of loops in 3dRNA_Oldlib and 3dRNA_Lib1. In order to verify the influence of 3dRNA_Lib1 and 3dRNA_Lib2 template libraries on the prediction accuracy of 3dRNA, we will test 3dRNA on different test sets later. For a target RNA, 3dRNA can give assembled and optimized structures [28]. The assembled structure is assembled by using the 3D templates for each SSE of the target RNA and minimized by a gradient-descent algorithm to avoid atom clash. It can be further optimized by a simulated annealing Monte Carlo (SAMC) algorithm to give optimized structures. In the SAMC optimization process, a randomly chosen moveable element will be translated, rotated around a point, or rotated around an axis. Then, a set of conformations are sampled and clustered by using the k-means clustering algorithm according to their Root-Mean-Square-Deviation (RMSD) values from each other. Finally, the ranked top N predictions (top N optimized structures) are given by the centroid of each cluster which is determined and ranked by 3dRNAscore [25]. It is noted that in the following the 3D templates from each target RNA itself are removed during the prediction of this RNA unless otherwise specified. Our evaluation of the accuracy for 3D structure prediction is measured by RMSD. In the following, 3dRNA using 3dRNA_Oldlib, 3dRNA using 3dRNA_Lib1, 3dRNA using 3dRNA_Lib2 are often simplified as “3dRNA_Oldlib”, “3dRNA_Lib1”, and “3dRNA_Lib2”.

### 3.1 Benchmark in all RNAs (Test Set I)

**Fig. 5:**
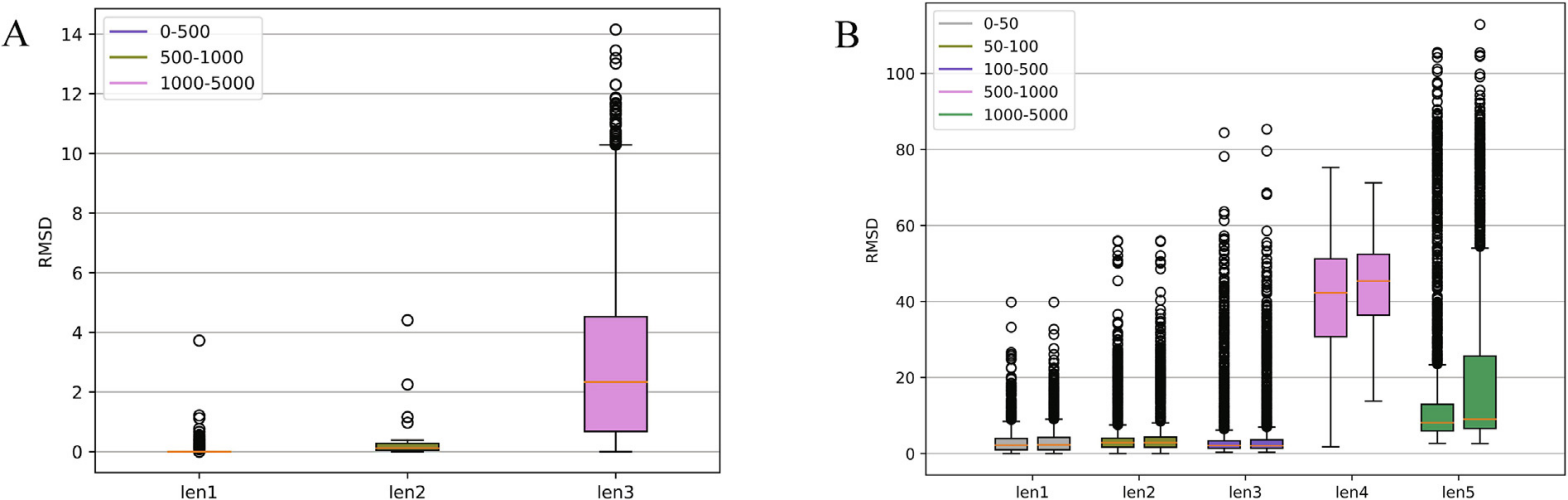
The relations between the lengths and RMSDs of the predictions for the RNAs in Test Set I. (A) The RMSDs of the RNAs under three different length ranges (len1, len2 and len3) when using “3dRNA_Lib1” with self-inclusion. The len1, len2 and len3 represent 0-500nt, 500-1000nt and 1000-5000nt, respectively. (B) The RMSDs of RNAs under five different length ranges (len1, len2, len3, len4 and len5) when using “3dRNA_Lib1” and “3dRNA_Lib2” with self-exclusion. The len1, len2, len3, len4 and len5 represent 0-50nt, 50-100nt, 100-500nt, 500-1000nt and 1000nt-5000nt, respectively. For each length range, the left and right boxes represent the RMSDs of the predictions of “3dRNA_Lib1” and “3dRNA_Lib2”, respectively.

In order to verify the correctness of our template library construction and test the overall prediction performance of 3dRNA with the new template libraries, all RNAs (6409 single-strand RNAs) are used as a test set (Test Set I). Since the optimization of 3dRNA is very time-consuming, only assembled structures are given here. We first use “3dRNA_Lib1” and “3dRNA_Lib2” to predict 3D structures of the RNAs in Test Set I with self-inclusion to see whether the SSEs of each RNA can find themselves as their templates in the 3D template libraries. The prediction results in “3dRNA_Lib1” are shown in Fig. 5A and our analysis shows that all RNA monomers can find themselves in the library as the final templates. The RMSDs of the predicted structures in relative to the native structures are within 15 Å. For the RNAs with lengths less than 1000nt, the predictions are basically near the native ones. For the RNAs longer than 1000nt, the average RMSD with the native ones is about 2 Å.

Fig. 5B shows the predictions of “3dRNA_Lib1” and “3dRNA_Lib2” with self-exclusion on Test Set I. Generally speaking, the performance of “3dRNA_Lib1” is better than “3dRNA_Lib2”. For “3dRNA_lib1”, the average RMSD is 2 Å for 0 ~ 50nt RNA and about 3 Å for 50 ~ 500nt RNAs. For 500 ~ 1000nt RNAs, due to their complex structures, they are very different from the native ones with an average RMSD of 40 Å. The RMSD distributions of the predictions of “3dRNA_lib1” and “3dRNA_lib2” on Test Set I with self-inclusion and self-exclusion are shown in Supplementary Figure S1 and Supplementary Figure S2, respectively.

### 3.2 Improvement of the predictions for short RNAs

In order to test the performance of the improved template library in predicting short RNAs, we analyzed 32 RNA used by 3dRNA-2.0 [23], [28], which is named as Test Set II. The lengths of RNAs in Test Set II are between 12nt and 110nt, including simple hairpin like 1ZIH_0 and also complex junction like 1Z43_0. In order to be more representative, the similarity between any two sequences is less than 50%. Supplementary Table S1 shows the prediction results of 3dRNA using different template libraries 3dRNA_Oldlib, 3dRNA_Lib1 and 3dRNA_Lib2. We mainly compare the RMSDs of predicted and native structures.

In general, the average RMSD of the assembled structures is reduced from 5.19 Å of “3dRNA_Oldlib” to 3.19 Å of “3dRNA_Lib1”, and that of the optimized structures is reduced from 4.16 Å of “3dRNA_Oldlib” to 3.03 Å of “3dRNA_Lib1”. In particular, the poor predictions for 1NYI_1, 28SP_0, 1J1U_0, 1N8X_0, and 1Z43_0 with “3dRNA_Oldlib” are obviously improved with “3dRNA_Lib1”. The prediction accuracies of “3dRNA_Lib1” and “3dRNA_Lib2” are similar in this case.

### 3.3 Improvement of the predictions for RNAs difficult to predict

In order to show the advantages of the new template library more intuitively, we have collected some RNAs that are very difficult to predict using “3dRNA_Oldlib” and name them as Test Set III. This test set includes 21 RNAs of different types and with lengths between 28nt to 158nt. The detailed description of them is shown in Supplementary Table S2. We shall compare the prediction accuracies of 3dRNA using the new libraries (3dRNA_Lib1 and 3dRNA_Lib2) with that using 3dRNA_Oldlib and that of RNAComposer. For comparing with the old library, both assembled and optimized structures are used. For comparing with RNAComposer, only optimized structures are used since we can only obtain optimized structures for the latter.

**Fig. 6:**
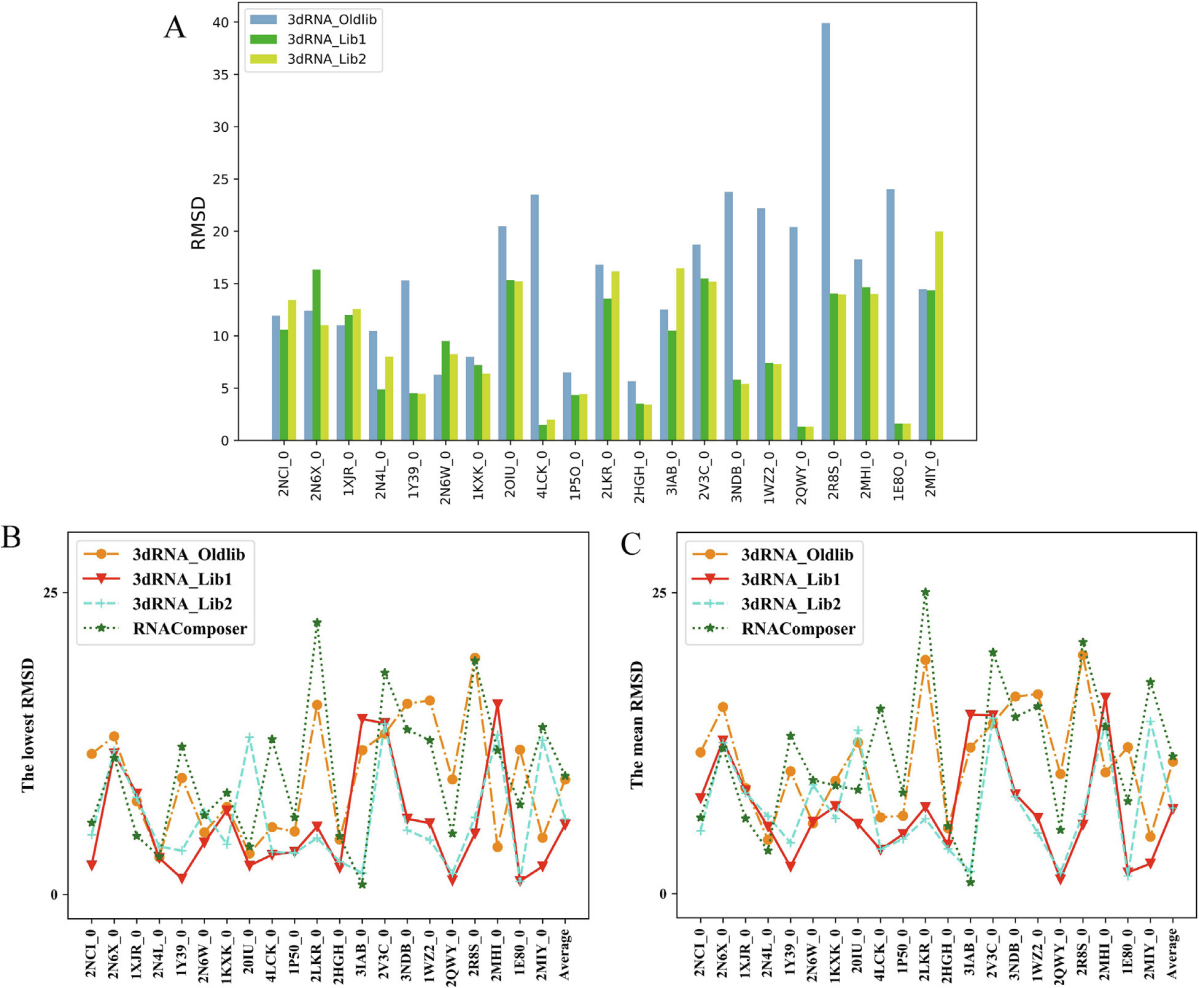
Comparison of the prediction accuracies (RMSDs) of 3dRNA using the new libraries with 3dRNA using old library and RNAComposer. (A) Comparison of the RMSDs of “3dRNA_Lib1” and “3dRNA_Lib2” with “3dRNA_Oldlib” for assembled structures. (B) and (C) Comparison of the RMSDs of “3dRNA_Lib1” and “3dRNA_Lib2” with “3dRNA_Oldlib” and RNAComposer for optimized structures. The lowest RMSD (B) and the mean RMSD (C) of the top 5 optimized structures of each RNA are used.

Fig. 6A shows a comparison of 3dRNA under three different template libraries 3dRNA_Oldlib, 3dRNA_Lib1 and 3dRNA_Lib2 for assembled structures. We find that the new libraries (3dRNA_Lib1 and 3dRNA_Lib2) give small RMSDs for 19 out of 21 cases than 3dRNA_Oldlib and the mean RMSDs of “3dRNA_Oldlib”, “3dRNA_Lib1” and “3dRNA_Lib2” are 16.24 Å, 8.96 Å and 9.54 Å, respectively. In general, “3dRNA_Lib1” overperforms “3dRNA_Oldlib” significantly and “3dRNA_Lib1” yields similar accuracy as “3dRNA_Lib2”.

In order to have a fair comparison with RNAComposer, both the lowest RMSD (Fig. 6B) and the mean RMSD (Fig. 6C) of the top 5 optimized structures are used as in RNA-Puzzles. As show in Fig. 6B and Fig. 6C, our model “3dRNA_Lib1” gives the best predictions for Test Set III with average value 5.77 Å for the lowest RMSDs of and 7.03 Å for the mean RMSDs. In addition, 3dRNA with the new libraries give the lowest RMSD for 18 out of 21 cases than 3dRNA_Oldlib or RNAComposer. The detail results of 3dRNA in the three template libraries and RNAComposer are given in Supplementary Table S3.

### 3.4 Improvement of predictions for long RNAs

**Fig. 7:**
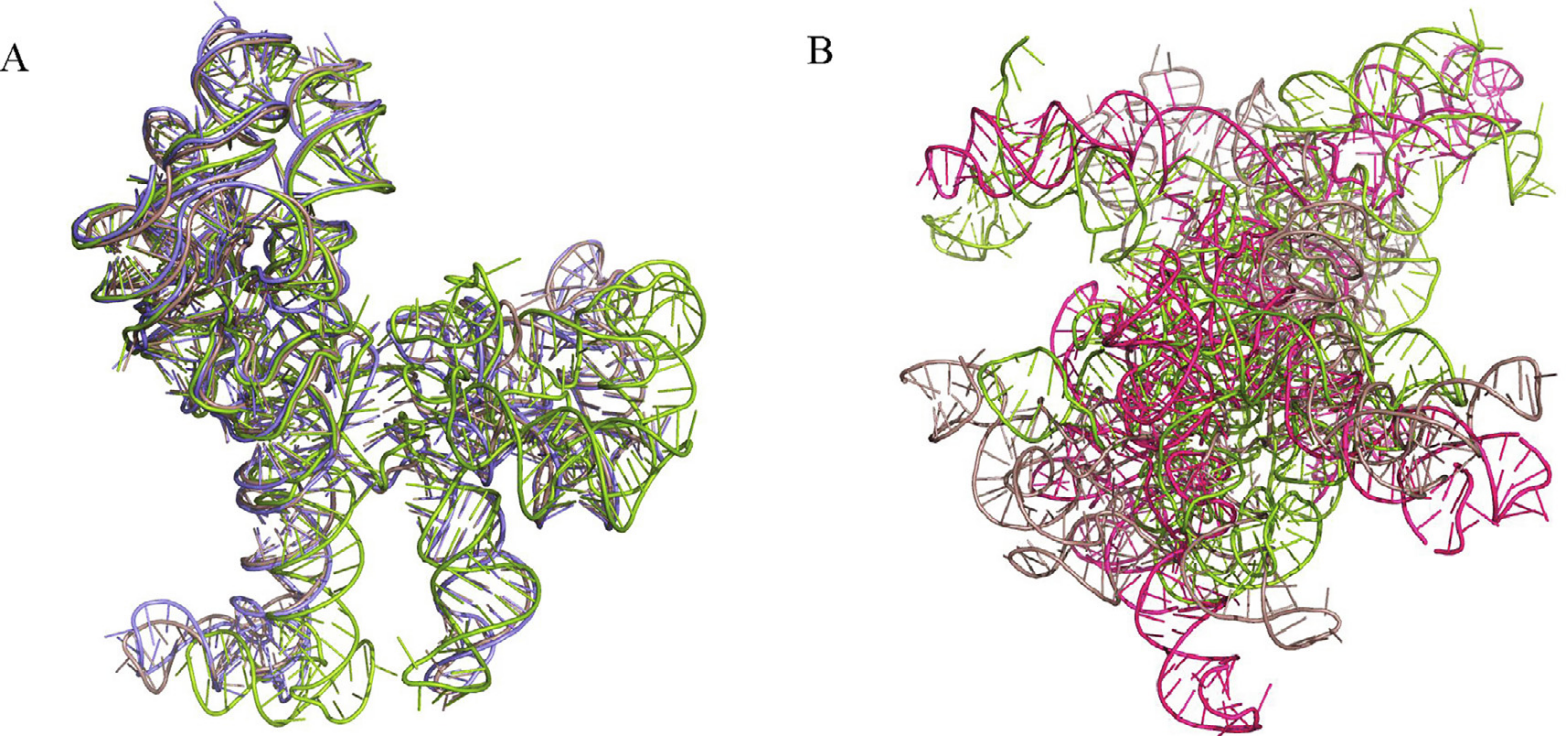
Predicted and native 3D structures of a riboswitch (PDB id: 1FFZ). (A) The native (pink), assembled (green) and optimized (blue) structures by “3dRNA_Lib1”. (B) The native structure (pink), the assembled structure (green) by “3dRNA_Oldlib” and the optimized structure (red) by RNAComposer. (For interpretation of the references to color in this figure legend, the reader is referred to the web version of this article.)

As mentioned above, at present, it is quite difficult to predict the 3D structures of long RNAs. We found that 3dRNA with the new template library can significantly improve the accuracy of predictions for long RNAs. We selected 5 large riboswitches, ranging from 500nt to 3000nt and name them as Test Set IV to show this. Among the five RNAs, 3dRNA using 3dRNA_Oldlib can only predict the structures of 1C2W_0 and 1FFZ_0 due to the limitation of the library. Not only can “3dRNA_Lib1” and “3dRNA_Lib2” predict the 3D structures of the five RNAs, but the assembled structures of four of them have good performance with the accuracy within 15 Å. RNAComposer can only predict one of the five RNAs within 500nt (1FFZ_0). Table 1 lists the RMSDs of the assembled and optimized structures of these riboswitches. The prediction results of 1FFZ_0 are compared in Fig. 7. It can be seen that the optimized structure of “3dRNA_Lib1” is very close to the native one, but the assembled structure deviates from the native one due to the orientation problem of the four-branch junction. The overall structures predicted by “3dRNA_Oldlib” and RNAComposer are very different from the native one.

**Table 1:** The prediction results of 3dRNA and RNAComposer for 5 large riboswitches.

PDB	Length (nt)	3dRNA_oldlib		3dRNA_lib1		3dRNA_lib2		RNAComposer
		ass	opt	ass	opt	ass	opt	opt
1C2W_0	2904	92.68	92.67	97.65	85.41	82.73	85.93	**\**
1FFZ_0	496	46.12	45.87	5.03	2.76	5.13	12.15	39.07
1I94_0	1514	**\**	**\**	6.97	7.21	26.34	16.10	**\**
1J5A_0	2774	**\**	**\**	13.99	16.00	14.34	32.36	**\**
6QUL_0	2795	**\**	**\**	8.71	6.00	9.31	6.81	**\**

### 3.5 Improvement of RNA-Puzzles predictions

Here 12 challenges of RNA-Puzzles [35], [36], [37] are predicted by 3dRNA with different template libraries. The native structures of puzzle6, puzzle13, puzzle14 and puzzle17 have broken chains and are completed accordingly. Supplementary Table S4 shows the sequences and secondary structures for 3dRNA inputs, in which the secondary structures are calculated from the corresponding native structures through X3DNA, and we also retain all non-canonical base pairs.

**Fig. 8:**
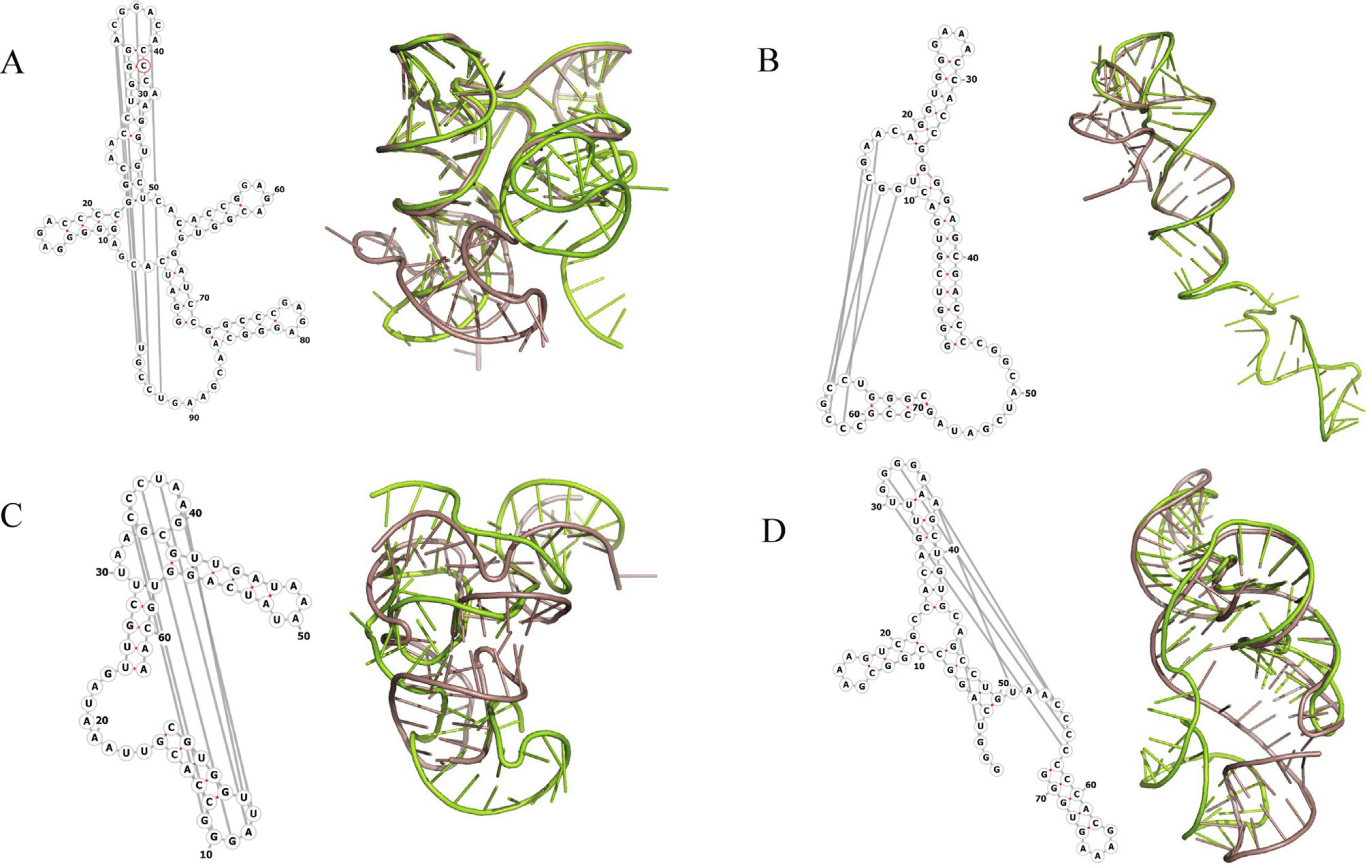
2D and 3D Structures of (A) puzzle8, (B) puzzle13, (C) puzzle17 and (D) puzzle18 which are difficult to predict by “3dRNA_Lib1”. The pink color shows the experimentally determined structures, the green color shows the assembled structures of “3dRNA_Lib1”. The 2D and 3D structures are generated using Forna and PyMOL [39] (http://www.pymol.org/), respectively. (For interpretation of the references to color in this figure legend, the reader is referred to the web version of this article.)

Supplementary Table S5 shows performances of 3dRNA using different libraries and RNAComposer. These 12 RNAs have lengths between 41nt and 188nt and are considered as RNAs with relatively complex structures. For “3dRNA_Oldlib”, the differences between all predicted structures and native structures are very large and the average RMSD of assembled and optimized structures are about 20.12 Å and 16.76 Å, respectively. However, for “3dRNA_Lib1”, the prediction accuracies of most puzzles are significantly improved in relative to “3dRNA_Oldlib”. The average RMSD decreases to 10.30 Å for assembled structures and 7.66 Å for optimized structures and both are improved by about 50%. The performance of “3dRNA_Lib2” is slightly inferior to that of “3dRNA_Lib1”. Fig. 8 shows the RNAs that their assembled structures by “3dRNA_Lib1” are poor predictions. We found that for puzzle6 the template of the four-way junction in it could not be found in the template library, which affected the global structure, while for puzzle8, puzzle13, puzzle17 and puzzle18 all are due to the lack of suitable templates for open loops in 3dRNA_Lib1.

We also used RNAComposer to predict these 12 puzzles with the same 2D structures. Overall, the prediction accuracy of RNAComposer is similar to that of “3dRNA_Oldlib”. The puzzle5, puzzle6, puzzle8, puzzle13, puzzle14, and puzzle21 have very poor prediction results due to the lack of templates for the corresponding loop regions in FRABASE [18], [19], and Puzzle7 has considerable RMSD due to the replacement of the orientation of the helix connected to multi-branch junction.

### 3.6 Running time of assembly

**Fig. 9:**
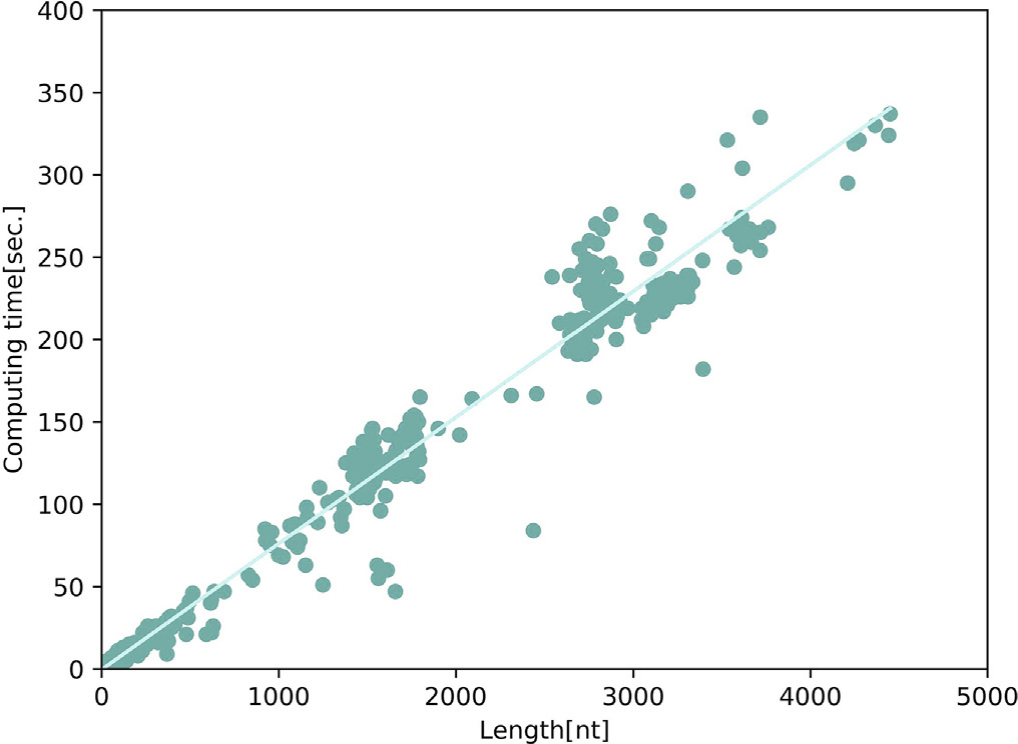
Running times of assembling RNA structures by 3dRNA.

In order to estimate the consuming time of assembling 3D structure by 3dRNA, the running times of 588 RNAs in Test Set I are given in Fig. 9. These RNAs are randomly selected from Test Set I according to chain length. Fig. 9 shows that the running times of assembling 3D structures increase linearly with RNA lengths roughly. The running times of the RNAs of less than 500nt are within 30 sec. For an RNA with length of 4000nt, the running time is about 350sec.

## 4 Summary

We have improved the 3D template library of 3dRNA. With the number of solved RNA 3D structures increasing in the PDB, the templates of 3dRNA will be continuously enriched. Comparing with the previous template library, the number of the templates in the improved template libraries is increased by about ten times. Comparing with “3dRNA_Oldlib”, the prediction accuracy of “3dRNA_Lib1” and “3dRNA_Lib2” are improved considerably, not only for small molecules but also for RNAs with complicated or large structures.

In order to ensure the synchronization of the template library of 3dRNA with newly added RNAs in PDB, we will automatically monitor the PDB database regularly in the later to add the SSEs of new RNAs into the template library. In future, we will also intend to enlarge the number of open loops to improve the prediction accuracy of 3dRNA since the missing of open-loop templates affects the prediction accuracy of RNA too.

Availability and Implementation

The web server of 3dRNA with the new template library is available at http://biophy.hust.edu.cn/new/3dRNA and the validation data can also be downloaded at the web server.

## CRediT authorship contribution statement

**Yi Zhang:** Methodology, Investigation, Formal analysis, Visualization, Software, Validation, Writing - original draft. **Jun Wang:** Methodology, Software, Validation. **Yi Xiao:** Conceptualization, Supervision, Methodology, Funding acquisition.

## Declaration of Competing Interest

The authors declare that they have no known competing financial interests or personal relationships that could have appeared to influence the work reported in this paper.
