# Supplementary material for: 3dRNA: Building RNA 3D structure with improved template library
Source: Comput Struct Biotechnol J. 2020 Aug 28;18:2416–23. doi: 10.1016/j.csbj.2020.08.017 (PMC7508704; doi:10.1016/j.csbj.2020.08.017)
Supplement: Supplementary data 1 [file mmc1.pdf]

Supplementary Materials

3dRNA: Building RNA 3D structure with improved template library

Yi Zhang[1], Jun Wang[1], Yi Xiao[2]

Institute of Biophysics, School of Physics, Huazhong University of Science and Technology, Wuhan 430074, Hubei, China

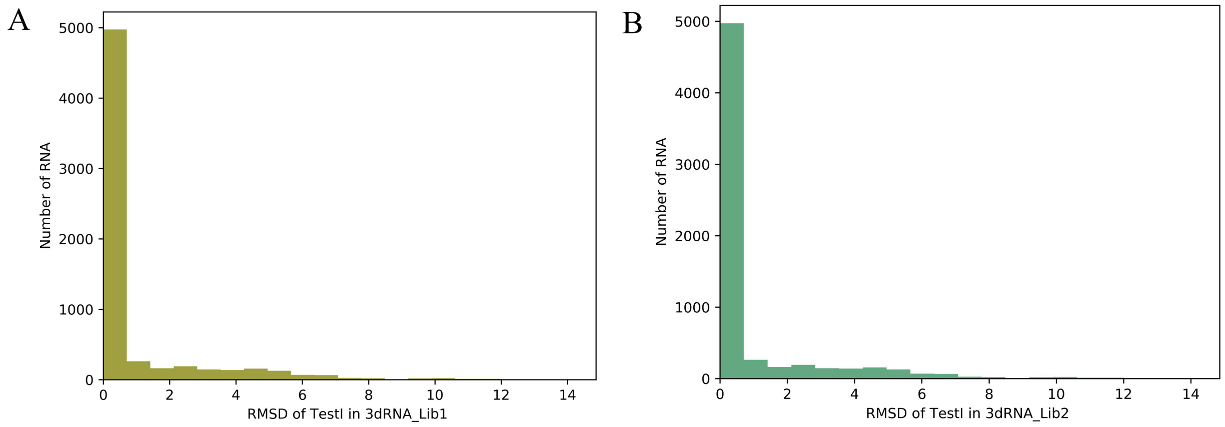

Supplementary Figure 1 - Prediction performance of 3dRNA in TestI with RNA self-inclusion in 3dRNA_Lib1 (A) and 3dRNA_Lib2 (B).

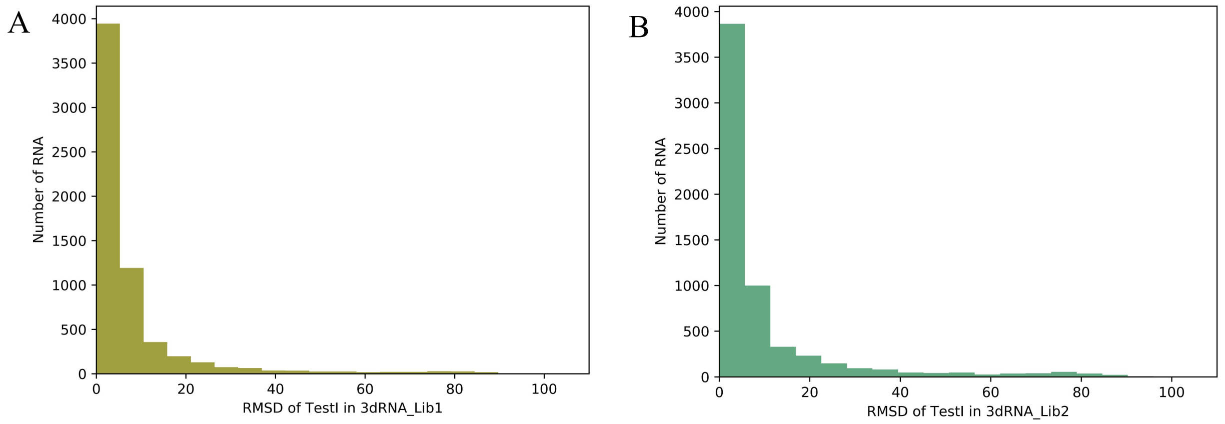

Supplementary Figure 2 - Prediction performance of 3dRNA in TestI with self-exclusion in 3dRNA_Lib1(A) and 3dRNA_Lib2(B).

---

[1] These authors made equal contribution.

[2] Corresponding author (yxiao@hust.edu.cn).

Supplementary tables

Supplementary Table 1 - Comparison of performance of 3dRNA in Test II from different Template Libraries

RNA	Length	3dRNA_Oldlib		3dRNA_Newlib			
				3dRNA_Lib1		3dRNA_Lib2	
		ass	opt	ass	opt	ass	opt
1C0O_0	14	1.81	1.67	3.32	1.59	3.32	1.33
1ZIH_0	12	1.67	2.19	1.79	1.26	1.89	1.34
1NEM_0	23	3.65	3.68	1.56	2.71	1.65	2.71
1K6G_0	22	3.62	2.36	3.76	2.09	3.76	2.10
2GIP_0	28	2.53	1.99	1.43	1.68	1.07	1.65
1NYI_1	24	10.28	7.22	1.47	0.84	1.38	0.85
2KVN_0	17	3.66	2.38	2.58	2.63	2.58	2.53
28SP_0	28	6.71	2.39	2.21	2.47	2.11	2.62
1HS4_0	13	3.37	1.23	3.47	2.96	4.59	2.95
1SLP_0	19	3.37	2.91	0.80	2.77	0.80	2.66
1F6X_0	27	2.02	2.75	3.14	2.42	3.88	3.03
1TXS_0	38	3.33	3.08	1.99	2.43	2.00	1.97
1EBQ_0	29	3.23	2.98	3.48	3.17	3.33	3.10
1KPZ_0	28	3.80	3.86	2.51	2.30	2.51	2.29
2ES5_0	23	2.06	1.91	2.03	1.50	2.03	2.91
2TOB_0	20	3.52	2.76	2.93	2.06	2.93	2.00
1J1U_0	74	7.34	5.73	3.25	2.49	2.37	2.85
1OW9_0	23	3.67	2.25	2.42	2.47	2.29	2.36
1N8X_0	36	7.93	2.76	2.95	1.84	3.12	3.00
2FDT_0	36	2.49	2.77	2.68	2.77	3.21	2.78
2TPK_0	36	13.09	6.27	17.79	5.84	17.67	5.43
2IXY_0	27	3.24	2.22	3.39	2.61	3.39	3.31
1JO7_0	31	4.29	3.84	4.62	3.34	6.46	5.64
1ZC5_0	41	2.94	2.80	1.74	2.57	1.74	2.36
1NA2_0	30	5.62	4.21	3.64	4.45	3.64	4.98
1E95_0	36	13.03	13.03	13.45	3.03	13.45	3.12
1Z43_0	101	21.87	5.43	4.26	1.85	3.66	2.00
1LVJ_0	31	6.12	6.34	8.80	5.79	9.53	3.69
1YNC_0	31	5.36	3.84	3.19	3.59	3.19	3.21
2KX8_0	42	4.63	3.63	3.45	4.20	3.45	4.17
1KXK_0	70	7.99	7.23	7.18	6.96	6.38	4.13
1XJR_0	47	11.01	9.03	11.98	8.34	12.57	8.03
Average	33	5.60	3.96	4.16	3.03	4.24	3.03

Supplementary Table 2 - The detail descriptions of RNA in Test Set III

PDB	Strand length (nt)	Structure style
2NCI_0	28	Hairpin, internal loop(has)
2N6X_0	43	Hairpin, internal loop
1XJR_0	47	Hairpin, internal loop(has), open loop
2N4L_0	53	Hairpin, internal loop(has)
1Y39_0	58	Hairpins, internal loop, 3-way junction(has)
2N6W_0	68	Hairpin, internal loop(has)
1KXK_0	70	Hairpin, internal loop(has), open loop
2OIU_0	71	Hairpins, internal loop, 3-way junction(has), open loop
4LCK_0	75	Hairpins(has), 4-way junction, open loop
1P5O_0	77	Hairpin, internal loop
2LKR_0	111	Hairpins, internal loop, 3-way junction, open loop
2HGH_0	55	Hairpins, internal loop, 3-way junction
3IAB_0	46	Hairpins, internal loop
2V3C_0	96	Hairpins, internal loop, 3-way junction, open loop
3NDB_0	136	Hairpins, internal loop, 3-way junction
1WZ2_0	88	Hairpins(has), 5-way junction, open loop
2QWY_0	55	Hairpins(has), internal loop, open loop
2R8S_0	159	Hairpins, internal loop(has), 3-way junction, open loop
2MHI_0	53	Hairpins, internal loop, 3-way junction(has)
1E8O_0	50	Hairpins, 3-way junction
2MIY_0	59	Hairpins ,open loop(has)

Supplementary Table 3 - The predicted RMSD of RNAcomposer and 3dRNA in three template libraries in Test Set III

PDB	3dRNA_Lib1			3dRNA_Lib2			3dRNA_Oldlib			RNAComposer	
	ass	opt		ass	opt		ass	opt		opt	
		lowest	mean		lowest	mean		lowest	mean	lowest	mean
2NCI_0	10.58	2.39	7.92	13.42	4.94	5.21	11.92	11.63	11.74	5.94	6.32
2N6X_0	16.33	11.66	12.71	11.03	11.90	12.53	12.40	13.09	15.51	11.30	12.11
1XJR_0	11.98	8.34	8.57	12.57	8.03	8.35	11.01	7.71	8.77	4.84	6.25
2N4L_0	4.87	3.01	5.56	8.01	3.96	6.43	10.47	3.08	4.48	3.14	3.58
1Y39_0	4.51	1.31	2.24	4.45	3.62	4.22	15.28	9.64	10.17	12.24	13.11
2N6W_0	9.48	4.28	5.97	8.24	6.83	9.04	6.28	5.14	5.84	6.56	9.43
1KXK_0	7.18	6.96	7.26	6.38	4.14	6.24	7.99	7.24	9.38	8.42	8.98
20IU_0	15.31	2.38	5.80	15.21	13.01	13.58	20.47	3.38	12.56	3.96	8.62
4LCK_0	1.48	3.28	3.65	1.97	3.52	3.71	23.50	5.57	6.34	12.85	15.36
1P50_0	4.34	3.51	4.94	4.42	3.42	4.53	6.47	5.21	6.47	6.37	8.39
2LKR_0	13.54	5.61	7.20	16.16	4.66	6.22	16.80	15.69	19.41	22.51	25.05
2HGH_0	3.50	2.20	4.06	3.42	2.72	3.72	5.64	4.54	5.44	4.82	5.60
3IAB_0	10.50	14.52	14.86	16.46	1.78	1.85	12.49	11.94	12.14	0.82	0.95
2V3C_0	15.47	14.16	14.81	15.18	14.12	14.59	18.72	13.30	14.14	18.36	20.05
3NDB_0	5.79	6.25	8.26	5.41	5.32	8.06	23.75	15.79	16.37	13.65	14.67
1WZ2_0	7.39	5.90	6.30	7.29	4.51	5.03	22.18	16.05	16.57	12.76	15.56
2QWY_0	1.31	1.16	1.18	1.31	1.74	1.75	20.40	9.52	9.94	5.04	5.30
2R8S_0	14.04	5.03	5.72	13.95	6.39	6.60	39.88	19.59	19.81	19.36	20.90
2MHI_0	14.64	15.73	16.28	14.01	13.19	14.12	17.30	3.93	10.07	11.92	13.85
1E80_0	1.60	1.10	1.76	1.59	1.08	1.47	24.02	11.98	12.17	7.43	7.68
2MIY_0	14.36	2.31	2.50	19.98	12.77	14.32	14.45	4.69	4.73	13.85	17.58
Average	8.96	5.77	7.03	9.54	6.25	7.11	16.26	9.51	10.97	9.81	11.40

Supplementary Table 4 - The sequence and secondary structure of RNA Puzzles (indicated by dotted brackets)

PDB	sequence/2D structure
Puzzle 3	CUCUGGAGAGAACCGUUUAAUCGGUCGCCGAAGGAGCAAGCUCUGCGGAAACGCAG<br>AGUGAAACUCUCAGGCAAAAGGACAGAG<br>((((((((. (. ((((. . . . . .))))) ((((. . (((((. (((((((((. . . .)))))<br>)))) .) .))) .))))) . . . .))))))))
Puzzle 4	GGCUUAUCAAGAGAGGUGGAGGGACUGGCCCGAUGAAACCCGGCAACCACUAGUCU<br>AGCGUCAGCUUCGGCUGACGCUAGGCUAGUGGUGCCAAUUCCUGCAGCGGAAACGU<br>UGAAAGAUGAGCCA<br>((((((((. . . . (. (((((((((. [[[[ ))) . . .))))))) (((. . ((((((((((<br>(((((((((. . . .) .))) . . . ( ]]]] (((((. . . .)))<br>)) . .)))))))))) .
Puzzle 5	GGUUGGGUUGGGAAGUAUCAUGGCUAAUCACCAUGAUGCAAUCGGGUUGAACACU<br>UAAUUGGGUUAAAACGGUGGGGGACGAUCCCGUAACAUCCGUCCUAACGGCGACAG<br>ACUGCACGGCCCUGCCUCUUAGGUGUGUCCAAUGAACAGUCGUUCCGAAAGGAAGC<br>AUCCGGUAUCCCAAGACAAUC<br>((((((((((((. . (((((((((((. . [[[ )))))))))))) . { ((((((((((((((<br>. . .) ((((((. { . (((((. (((. . .]]]))) . . . . . .) .)))) ((. . . })) . . . . . [[<br>[[[[. . .)))))) ((((. . .))))))))))))))) . } .]]]]] . ((((. . . .))))) . .<br>] .))))) . . .)))))))))))))
Puzzle 6	CGGCAGGUGCUCCCGACCCUGCGGUCGGGAGUUAAAAGGGAAGCCGGUGCAAGUCC<br>GGCACGGUCCCGCCACUGUGACGGGGAGUCGCCCCUCGGGAUGUGCCACUGGCCAA<br>GGGCCGGGAAGGCGGAGGGGCGGCGAGGAUCCGGAGUCAGGAAACCUGCCUGCCG<br>((((((((. (((. (((. . . . .)) .)))) (. . .) (((. . . (((((({ . .) < {))<br>))) [[((. . . . . .)) ((((} . } (((((((((((((. ((. . . . . (. ((((((((((. .<br>.)))))))) . .))))))) .)))))))) . . .))))))) . >]]))))) . . .))))))))))))
Puzzle 7	GCGCUGUGUCGCAAUCUGCGAAGGGCGUCGUCGGCCCAAGCGGUAGUAAGCAGGGA<br>ACUCACCUCCAAUGAAACACAUUGUCGUAGCAGUUGACUACUGUUAUGUGAUUGGU<br>AGAGGCUAAGUGACGGUAUUGGCGUAAGCCAAUACCGCAGCACAGCACAAGCCCGC<br>UUGCGAGAUUACAGCGC<br>(((((((((((. ([. (((((. . (((((. . . . .))))) .))))) .) (((((([((((<br>. ((((((((((((. . . . .))))) . . (((((((. . . . .))))))) . . . .))))) .<br>))) ((( ] . ((((((((((((((. . . .))))))))))) . .)))))))) . . . .)))))))<br>)))]))))) .))))))))
Puzzle 8	GGAUCACGAGGGGGAGACCCCGGCAACCUGGGACGGACACCCAAGGUGCUCACACC<br>GGAGACGGUGGAUCCGGCCCGAGAGGGCAACGAAGUCCGU<br>(((((. . . . ((((. . . . .)))) (((. . (((((([[[[[. .))) .))) .))) . . ((((<br>(. . . . .))))))))))) (((((. . . . .))))) . . . . . .]]]]]
Puzzle 12	GAUCGCUGAACCCGAAGGGGCGGGGGACCCAGGGGGCGAAUCUCUUCCGAAAGGAA<br>GAGUAGGGUUACUCCUUCGACCCGAGCCCGUCAGCUAACCUCGCAAGCGUCCGAAG<br>GAGAA<br>. . . (((((. . (((. . .))) (((((((((. ((. . ((((((. . (((((((((. . . . .)))))<br>)))) ((((((. [[[[[[[[ )))))))))))))) . .) .))) .)))))))))))))) . . .]]]

	]]]] . .
Puzzle 13	GGGUCGUGACUGGCGAACAGGUGGGAAACCACCGGGGAGCGACCCCGGCAUCGAUA GCCGCCCGCCUGGGC<br>((((((((((([[[[. [. ((((((. . . .))))))))))))))))) . . . . ((((.]]]]] .)))))
Puzzle 14	CGUUGGCCCAGGAAACUGGGUGGAAGUAAGGCCCAUUGCACUCCGGGCCUGAAGCA ACGCU<br>(((((((((((. . . .)))))) . . . (. ((((((((. . . . .))))))))) .))))) . .
Puzzle 17	CGUGGUUAGGGCCACGUUAAAUAGUUGCUUAAGCCCUAAGCGUUGAUAAAUAUCAG GUGCAA<br>((((([[[[[[ ))))) . . . . (((((. . . ( ]]]]]] .) ((((((. . . .)))))))))))
Puzzle 18	GGGUCAGGCCGGCGAAAGUCGCCACAGUUUGGGGAAAGCUGUGCAGCCUGUAACCC CCCCACGAAAGUGGG<br>. . { . (((((((((. . . .)))) . (((((((. [[[[.) [ )))))) } .)))))] . .]]]] (((((. . . .)))))
Puzzle 21	CCGGACGAGGUGCGCCGUACCCGGUCACGACAAGACGGCGC<br>[[[[[. . . . . . ((((((( ] ( ]]]] . . . .) . . . . .)))))))

Supplementary Table 5 - The prediction accuracies of 13 RNA puzzles of 3dRNA and RNAComposer

RNA	Length	3dRNA_Oldlib		3dRNA_Lib1		3dRNA_Lib2		RNAComposer
		ass	opt	ass	opt	ass	opt	
Puzzle 3	84	11.99	12.16	3.40	4.00	3.74	12.34	13.35
Puzzle 4	126	11.13	9.02	3.22	2.53	3.26	5.61	10.48
Puzzle 5	188	27.58	26.47	2.70	2.46	2.60	2.49	27.49
Puzzle 6	167	24.67	24.43	24.01	13.50	24.53	15.02	28.11
Puzzle 7	185	30.12	22.08	5.65	17.25	5.54	16.77	28.88
Puzzle 8	96	24.92	21.82	13.72	1.51	13.74	5.85	20.06
Puzzle 12	117	24.62	18.48	1.77	3.36	2.34	4.74	15.58
Puzzle 13	71	31.18	12.48	32.18	14.88	32.43	12.99	13.28
Puzzle 14	61	10.62	11.40	12.00	6.95	12.09	7.59	17.08
Puzzle 17	62	17.14	18.18	13.82	13.27	16.80	14.68	11.21
Puzzle 18	71	13.24	12.71	10.20	10.94	10.26	10.49	14.84
Puzzle 21	41	14.28	11.94	1.04	1.30	1.00	1.43	10.21
Average	105	20.12	16.76	10.30	7.66	10.69	9.16	17.55
